# Identification of hsa_circ_0001445 of a novel circRNA-miRNA-mRNA regulatory network as potential biomarker for coronary heart disease

**DOI:** 10.3389/fcvm.2023.1104223

**Published:** 2023-03-14

**Authors:** PhongSon Dinh, JunHua Peng, ThanhLoan Tran, DongFeng Wu, ChauMyThanh Tran, ThiPhuongHoai Dinh, ShangLing Pan

**Affiliations:** ^1^ Departments of Pathophysiology, Guangxi Medical University, Nanning, China; ^2^ College of Medicine and Pharmacy, Duy Tan University, Danang, Vietnam; ^3^ Key Laboratory of Longevity and Ageing-Related Disease of Chinese Ministry of Education, Center for Translational Medicine and School of Preclinical Medicine, Guangxi Medical University, Nanning, China; ^4^ Department of Immunology and Pathophysiology, Hue University of Medicine and Pharmacy, Hue University, Hue, Vietnam; ^5^ Department of the Geriatric Cardiology, Guangxi Academy of Medical Sciences and the People's Hospital of Guangxi Zhuang Autonomous Region, Nanning, China; ^6^ Department of Neurosurgery, Hue University Hospital, Hue University of Medicine and Pharmacy, Hue University, Hue, Vietnam

**Keywords:** coronary heart disease, hsa_circ_0001445, circRNA-miRNA-mRNA network, diagnostic markers, bioinformatics analysis

## Abstract

**Objects:**

To evaluate the hsa_circ_0001445 level in peripheral blood leukocytes of patients with coronary heart disease (CHD) and its related clinical factors, and predict its circRNA-miRNA-mRNA regulatory network in CHD pathogenesis *via* bioinformatics analysis**.**

**Methods:**

Peripheral blood leukocytes were isolated from the whole blood samples of 94 CHD patients (aged 65.96 ± 9.78 years old) and 126 healthy controls (aged 60.75 ± 8.81 years old). qRT-PCR was used to quantify the expression level of circRNA and subsequently analyze its association with CHD clinical parameters. Via bioinformatics algorithm and GEO datasets, differential miRNA expression was evaluated using the Limma package. A miRNA-mRNA regulatory network was predicted by cyTargetLinker. ClusterProfiler was employed to perform functional enrichment analysis of the circRNA network to investigate its role in CHD pathogenesis.

**Results:**

The expression of hsa_circ_0001445 in peripheral blood leukocytes of CHD patients was downregulated compared with that of healthy controls. Positive correlations were evident between hsa_circ_0001445 expression level and the levels of hemoglobin, triglycerides, high- and low-density lipoprotein cholesterol. A significant negative correlation was also found between hsa_circ_0001445 expression level and age and the neutrophil level. Low expression of hsa_circ_0001445 exhibited a discriminatory ability between CHD patients and healthy controls with a sensitivity of 67.5% and a specificity of 76.6% (*p* < 0.05). By bioinformatics analysis, 405 gene ontology terms were identified. The Kyoto Encyclopedia of Genes and Genomes terms focused principally on the PI3K-Akt signaling pathway. hsa_circ_0001445 was associated with the expression of three miRNAs that may regulate 18 genes involved in KEGG processes: hsa-miR-507, hsa-miR-375–3p, and hsa-miR-942–5p.

**Conclusion:**

The hsa_circ_0001445 level in peripheral blood leukocytes may serve as a biomarker for CHD diagnosis. Our work on circRNA-miRNA-mRNA networks suggests a potential role for hsa_circ_0001445 in CHD development.

## Introduction

Coronary heart disease (CHD) is the most common heart condition worldwide and the leading cause of death of elderly men and women (1). Despite recent declines in developed countries, both CHD morbidity and mortality continue to increase rapidly in developing countries. Various factors are involved in CHD pathogenesis, including older age, dyslipidemia, obesity, psychological issues, hypercholesterolemia, diabetes, and family history (2–4). Currently, most efforts focus on biochemical tests, protein and gene based biomarkers to predict the incidence of CHD (5, 6). However, the ability of these factors in terms of early detection of CHD are still controversial (5). Recently, circular RNAs (circRNAs) have emerged as a novel potential non-invasive biomarker for diagnosis and prognostic of CHD patients.

CircRNAs are a kind of non-coding RNA that consists of continuous covalently closed loops without the 3′- and 5′ end like linear RNA, which enables it to resist degradation, and thus has relative conservation and stability (7). Recently, their functions and biological features have been extensively studied. They modify gene expression by serving as microRNA (miRNA) sponges that bind and inactivate certain miRNAs (8–10). Research on their roles in cardiovascular diseases has progressed rapidly. In particular, in-depth studies of the relationship between circRNA and CHD have provided effective tools to early diagnose CHD and by that reduced CHD mortality (5, 7, 11).

Recently, Vilades et al*.* (6) showed that the plasma levels of the circRNA hsa_circ_0001445 (hsa_circSMARCA5_013) were proportional to coronary atherosclerotic burden. hsa_circ_0001445 has been consistently detected in clinically relevant samples, including heart tissue (12), plasma (13), serum (14), and whole blood (15). In this study, we first investigated the levels of hsa_circ_0001445 in peripheral blood leukocytes (PBLs) of CHD patients from Guangxi, China (6), and analyzed its correlation with clinical characteristics. Next, we performed bioinformatics analyses to define a novel circRNA-miRNA-mRNA network involved in CHD. Finally, we conducted functional and pathway enrichment analyses of potentially relevant genes. Our findings may provide potential candidate for further studies on the pathogenesis of CHD.

## Methods

### Study population

The experimental group included 94 CHD patients aged 65.96 ± 9.78 years (58 men and 36 women) admitted to the Department of Cardiology of the People's Autonomous Hospital of Guangxi Zhuang from January 1 2019 to December 31 2020. All patients underwent coronary angiography (CAG), and those with stenoses ≥50% in at least one of the three main coronary arteries or their major branches (diameter ≥2 mm) were diagnosed with CHD. The exclusion criteria were diabetes mellitus, any other clinically acute or chronic inflammatory systemic disease, uncontrolled hypertension, liver or kidney dysfunction, endocrine disease, autoimmune disease, a malignancy, prior percutaneous coronary intervention (PCI) or coronary artery bypass grafting (CABG), and a history of CHD. The control group included 126 healthy subjects aged 60.75 ± 8.82 years (61 men and 65 women) recruited in the same period from the Second Affiliated Hospital of Guangxi Medical University. They were confirmed healthy after physical check-ups; none had a history of coronary atherosclerosis or microvascular disease. This study was conducted in accordance with the Declaration of Helsinki (1975) and was approved by the ethics committee of Guangxi Medical University (approval no. 2019-SB-060). All patients and controls gave written informed consent.

### Total RNA extraction

Total RNAs were extracted from PBLs of CHD patients and healthy controls using the SanPrep column microRNA extraction kits (Sangon Biotech, China); all samples were stored at −80°C.

### Reverse transcription polymerase chain reaction (RT-PCR)

RNA reverse transcription into cDNA was performed using 5× HiScript III qRT SuperMix kits (Vazyme Biotech, China) according to the manufacturer's instructions. One microgram of RNA and 4x gDNA wiper mix were incubated at 42°C for 2 min, then 5× HiScript III qRT SuperMix was added, followed by incubation at 37°C for 15 min and 85°C for 5 s. The products served as qRT-PCR templates.

### qRT-PCR analysis

The expression level of hsa_circ_0001445 was detected by qRT-PCR with Light Cycler 96 platform (Roche, USA), and Glyceraldehyde-3-phosphate dehydrogenase (hGAPDH) served as the internal standard for normalization. The specific primers were listed as follows: (hsa_circ_0001445) forward primer: 5′-TGGGCGAAAGTTCACTTAGAA-3′, reverse primer: 5′- CACATGTGTTGCTCCATGTCT-3′; (hGAPDH) forward primer: 5′- TGTTGCCATCAATGACCCCTT-3′, reverse primer: 5′-CTCCACGACGTACTCAGCG-3′ (6, 16). Each sample was performed in triplicate. The reaction conditions included of 40 cycles at 95°C for 3 min, 95°C for 10 s, and 60°C for 60s; the dissolution curves were obtained *via* one cycle at 95°C for 5 s, 60°C for 1 min, and 97°C for 1 s. The expression level of hsa_circ_0001445 was calculated using the 2^−ΔCt^ method relative to hGAPDH.

### Statistical analysis

Data were statistically analyzed using SPSS 22.0 (SPSS Inc. Chicago, IL, USA) and GraphPad Prism 8 (GraphPad Software, San Diego, California, USA). Continuous data were presented as mean ± standard deviation (means ± SD) if normally distributed, and otherwise as median (interquartile range). The circRNA expression levels between CHD and controls were compared using the Student's *t*-test (calculated by SPSS) to determine statistically significant difference between the means of two groups. Spearman's rho coefficient was used to assess the correlation between continuous variables. Logistic regression was used to assess relationships between various factors and the PBL levels of hsa_circ_0001445. A *p*-value < 0.05 was considered significant. Analysis of receiver operating characteristic (ROC) curves was performed to calculate the optimal area under the ROC curve (AUC) for evaluating the CHD diagnostic ability of hsa_circ_0001445.

### Differential miRNA expression and construction of the miRNA-mRNA regulatory network

miRNA expression levels of CHD patients were collected from the public Gene Expression Omnibus database (GEO) (https://www.ncbi.nlm.nih.gov/geo/) using the following criteria: peripheral blood cells from humans, ≥3 samples of patients and normal controls, and miRNAs expression in CHD patients. The relevant datasets were GSE105449 (GPL22949) (17) and GSE61741 (GPL9040) (18). There were 236 samples in total (136 control and 100 CHD) that met the above criteria. GSE IDs, the high-throughput data, and the annotated subject characteristics of the control and CHD groups were collected in a series matrix file and were analyzed using R software ver. 3.6.2.

R software (Version 3.6.2; R Foundation for Statistical Computing, Vienna, Austria) (https://www.r-project.org/), the Bioconductor software package (https://bioconductor.org/packages) (19), and the *limma* package (20) were used to analyze differential miRNA expression based on miRNA expression data. All *p*-values were corrected using the false discovery rate (FDR) correction toolkit. A *p*-value <0.05 and with an FDR < 0.05 for all GSE files (fold change >1) were considered significant. Then, Venn diagrams were framed to identify overlapping miRNAs among the predicted datas in order to determine which potential miRNAs were associated with hsa_circ_0001445 in CHD. miRNAs identified as significant were entered into Cytoscape ver. 3.6.1 (http://cytoscape.org/) and were used to establish a network. A regulatory network was predicted by cyTargetLinker (https://cytargetlinker.github.io/) using the targetscan-hsa-7 and miRTarBase-hsa-7 databases (21). Finally, a miRNA-mRNA network was constructed.

### Gene ontology (GO) and Kyoto encyclopedia of genes and genomes (KEGG) enrichment analyses

ClusterProfiler ver. 3.14.3 package in R software was used to analyze the gene ontology and signaling pathways. Genome-wide annotation for humans was based on mapping employing the Entrez gene identifiers; we used several methods to visualize and interpret the functional enrichment results. A *p*-value <0.05 served as a cut-off when determining significant enrichments of GO terms and KEGG pathways. That is, the more likely that the gene associated with the listed entry/pathway influences cellular life activities and warrants further research (22). Venny 2.1 diagram was used to improve predictive accuracies (*via* intersection) (https://bioinfogp.cnb.csic.es/tools/venny/). Finally, the interaction network of miRNAs with genes of the KEGG pathway was established.

## Results

### Expression of hsa_circ_0001445 in PBLs of CHD patients

**Figure 1:**
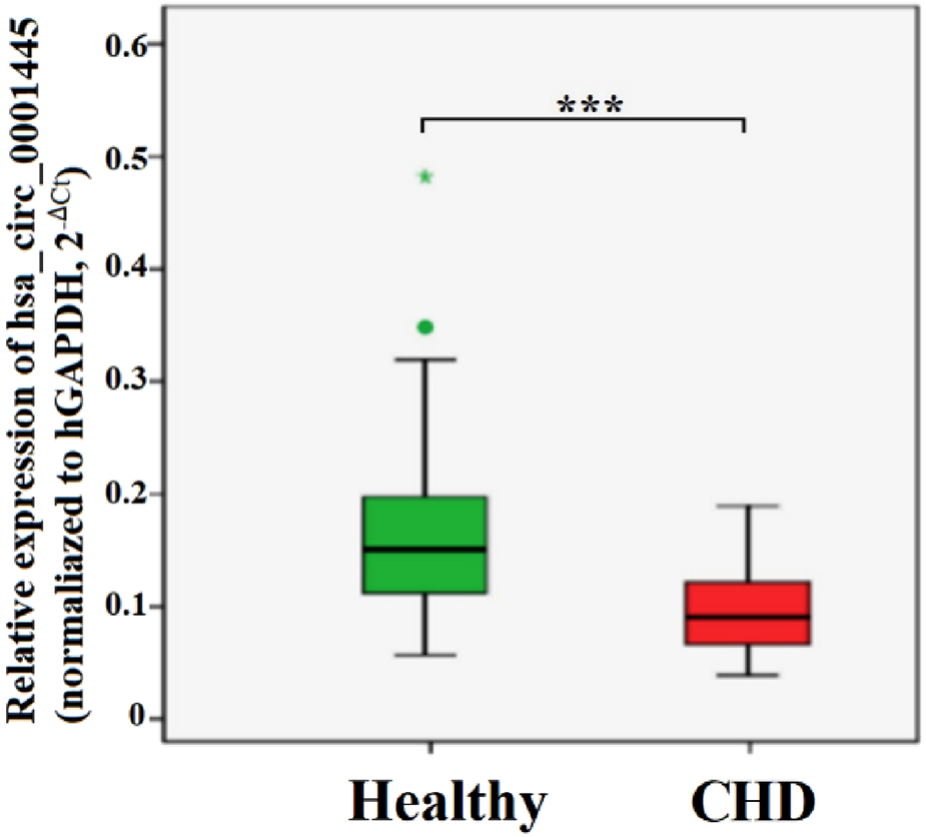
Expression levels of hsa_circ_0001445 in the healthy and CHD groups. *p* < 0.001.

The expression level of hsa_circ_0001445 in PBLs of CHD patients was significantly lower than that of healthy controls (0.640 ± 0.254 and 1.079 ± 0.453, respectively) (*p* < 0.001) (Figure 1).

### Clinical characteristics comparison between CHD patients and healthy controls groups

We compared the age, sex, blood parameters, fasting blood glucose levels (GLU), three renal function items (UREA, CREA, and UA), three liver enzymes (AST, ALT, GGT) and cholesterol levels of the two groups. As shown in Table 1, age, white blood cell count (WBC), and levels of hematocrit (HCT), neutrophils (NEU), red blood cells (RBCs), hemoglobin (HGB), platelets (PLT), alanine aminotransferase (ALT), aspartate transaminase (AST), glutamyl transpeptidase (GGT), glucose (GLU), total cholesterol (T-Cho), high-density lipoprotein cholesterol (HDL-C), and low-density lipoprotein cholesterol (LDL-C) significantly differed between the two groups (all *p* < 0.05).

**Table 1:** Clinical parameters of the CHD patients and healthy controls.

Clinical parameters	Healthy	CHD	*P* value
Age	60.746 ± 8.817	65.957 ± 9.780	<0.001
Sex (Male/Female)	61/65	58/36	0.051
WBC (10^9^/L)	6.2387 ± 1.475	6.877 ± 2.289	0.013
NEUTR (10^9^/L)	3.485 ± 1.115	4.234 ± 1.779	<0.001
RBC (10^12^/L)	4.815 ± 0.606	4.497 ± 0.620	<0.001
HGB (g/L)	138.218 ± 11.815	131.489 ± 15.641	<0.001
HCT	41.610 ± 3.199	39.485 ± 4.373	<0.001
PLT (10^9^/L)	251.108 ± 59.333	228.391 ± 64.189	0.008
AST (U/L)	20.246 ± 5.353	31.607 ± 35.559	0.001
ALT (U/L)	17.802 ± 6.516	29.122 ± 46.475	0.008
GGT (U/L)	26.776 ± 17.127	40.417 ± 40.246	0.001
Urea (mmol/L)	4.981 ± 1.130	5.351 ± 2.176	0.109
Crea (µmol/L)	79.429 ± 15.032	91.765 ± 115.907	0.239
UA (µmol/L)	329.659 ± 74.302	341.455 ± 129.966	0.464
GLU (mmol/L)	4.986 ± 0.502	4.677 ± 0.712	<0.001
T-Cho (mmol/L)	4.963 ± 1.038	4.457 ± 1.098	0.001
TG (mmol/L)	1.356 ± 0.70031	1.454 ± 0.981	0.416
HDL-C (mmol/L)	1.454 ± 0.398	1.110 ± 0.275	<0.001
LDL-C (mmol/L)	3.245 ± 0.852	2.642 ± 0.861	<0.001

### Relationships between PBL hsa_circ_0001445 expression level and clinical characteristics

Clinical parameters associated with hsa_circ_0001445 expression level in CHD patients were shown in Table 2. Its expression was negatively correlated with age and NEU but positively correlated with HGB, T-Cho, HDL-C, and LDL-C (*p* < 0.05).

**Table 2:** Correlations between hsa_circ_0001445 expression level and clinical parameters of CHD patients.

Factors	hsa_circ_0001445	
	*r*	*P* value
Age	−0.290	<0.001
WBC (10^9^/L)	−0.088	0.195
NEUTR (10^9^/L)	−0.137	0.043
RBC (10^12^/L)	0.142	0.036
HGB (g/L)	0.181	0.007
HCT	0.172	0.011
PLT (10^9^/L)	0.039	0.566
AST (U/L)	−0.103	0.136
ALT (U/L)	−0.021	0.781
GGT (U/L)	−0.102	0.143
GLU (mmol/L)	0.063	0.356
T-Cho (mmol/L)	0.249	<0.001
HDL-C (mmol/L)	0.284	<0.001
LDL-C (mmol/L)	0.293	<0.001

### Logistic regression analysis of associations between hsa_circ_0001445 expression level and clinical characteristics of CHD patients

We used multivariate logistic regression to explore whether hsa_circ_0001445 independently predicted CHD. Model 1 included NEU and HGB; model 2 included T-Cho, HDL-C, and LDL-C; and model 3 included NEU, HGB, T-Cho, HDL-C, and LDL-C. Based on the median 2^−*Δ*Ct^ values for hsa_circ_0001445, the CHD group was subdivided into those with low and high circRNA expression. In addition, based on the normal ranges and median values of NEU, HGB, T-Cho, and LDL-C, patients were subdivided into high and normal subgroups. The HDL-C values were used to define normal- and low-expression subgroups.

Low hsa_circ_0001445 expression level was an independent risk factor for CHD in all three models (Table 3).

**Table 3:** Logistic regression results.

	Model 1		Model 2		Model 3	
	OR (95% CI)	*P* value	OR (95% CI)	*P* value	OR (95% CI)	*P* value
hsa_circ_0001445	0.175	<0.001	0.158	<0.001	0.163	<0.001
	(0.096–0.319)		(0.081–0.306)		(0.084–0.318)	

### ROC curve analysis and the AUC of hsa_circ_0001445 for diagnosing CHD

**Figure 2:**
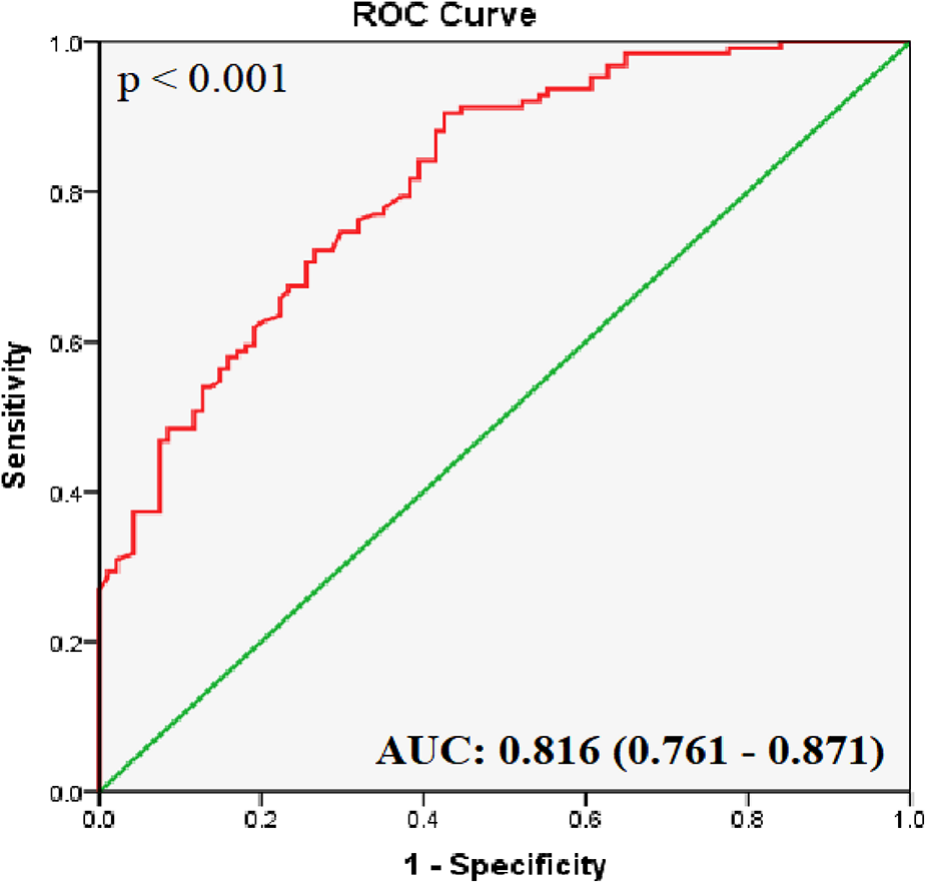
ROC curve analysis of hsa_circ_0001445. The AUC shows the diagnostic ability of hsa_circ_0001445 in terms of CHD (*p* < 0.001).

ROC curve analyses of the healthy and CHD groups showed that the AUC for hsa_circ_0001445 was 0.816 ± 0.028 (95% CI 0.761–0.871; *p* < 0.001; Figure 2). It had the highest *J* index [(Se + Sp − 1) = 0.441]; a 2^−*Δ*Ct^ value of 0.814 was thus chosen as the cut-off. The sensitivity was 67.5%, the specificity was 76.6%, and the likelihood ratio [Se/(1-Sp)] was 2.88.

### Differentially expressed miRNAs of CHD and the predicted miRNA-mRNA regulatory networks

We found 132 differentially expressed miRNAs in CHD, including 79 that were upregulated and 53 that were downregulated in GSE105449 and GSE61741 dataset; all met the cutoff criteria of abs (log_2_FC) >1 and *p* < 0.05 (Supplementary Table S1). The top 10 up- and downregulated miRNAs are listed in Tables 4, 5.

**Table 4:** Top 10 DmiREs from GSE105449.

Up-regulated miRNA	log2FC	*P* value	Down-regulated miRNA	log2FC	*P* value
hsa-miR-124-3p	1.344234242	0.008875452	hsa-miR-376a-3p	−1.251881245	0.040091924
hsa-miR-1305	1.240584333	0.001566456	hsa-miR-186-5p	−1.197982753	0.007514646
hsa-miR-1288-3p	1.175015237	0.016246456	hsa-miR-30b-5p	−1.178711234	0.004173543
hsa-miR-542-3p	1.174828635	0.008972344	hsa-miR-338-3p	−1.156623434	0.029673451
hsa-miR-1202	1.162524856	0.016565743	hsa-miR-326	−1.146023456	0.020356346
hsa-miR-378a-3p	1.128869631	0.031397457	hsa-miR-29c-5p	−1.118398224	0.022234453
hsa-miR-27b-3p	1.116581263	0.021416462	hsa-miR-548am-5p	−1.108357546	0.010322435
hsa-miR-139-3p	1.104053912	0.002214564	hsa-miR-29c-3p	−1.104433632	0.005536363
hsa-miR-520e	1.055113965	0.021147573	hsa-miR-17-5p	−1.094272355	0.007023452
hsa-miR-644a	1.034448666	0.005345673	hsa-miR-335-3p	−1.088737452	0.006563467

**Table 5:** Top 10 DmiREs from GSE61741.

Up-regulated miRNA	log2FC	*P* value	Down-regulated miRNA	log2FC	*P* value
hsa-miR-375-3p	2.055179668	3.41 × 10^−8^	hsa-miR-31-3p	−1.67133085	9.10 × 10^−8^
hsa-miR-142-3p	1.911637447	5.52 × 10^−7^	hsa-miR-1283	−1.632062369	8.10 × 10^−7^
hsa-miR-29c-3p	1.850096182	4.86 × 10^−8^	hsa-miR-200a	−1.60499372	3.77 × 10^−7^
hsa-miR-1258	1.846107258	1.34 × 10^−7^	hsa-miR-515-5p	−1.467324166	4.04 × 10^−8^
hsa-miR-302b-3p	1.789940606	2.09 × 10^−6^	hsa-miR-1245a	−1.409488453	3.83 × 10^−6^
hsa-miR-1468-5p	1.711611027	1.76 × 10^−6^	hsa-miR-155-3p	−1.376257877	1.03 × 10^−5^
hsa-miR-520c-3p	1.647465652	7.74 × 10^−7^	hsa-miR-488-5p	−1.368851066	3.19 × 10^−7^
hsa-miR-204-5p	1.643870075	7.73 × 10^−7^	hsa-miR-21-3p	−1.324586005	0.000113238
hsa-miR-609	1.621093043	2.97 × 10^−5^	hsa-miR-519b-5p	−1.275137605	9.00 × 10^−6^
hsa-miR-132-5p	1.603354363	1.14 × 10^−7^	hsa-miR-545-3p	−1.217793064	8.00 × 10^−5^

The Circbank and Circinteractome databases were used to predict interactions between hsa_circ_0001445 and miRNAs. We identified 26 interacting pairs.

**Figure 3:**
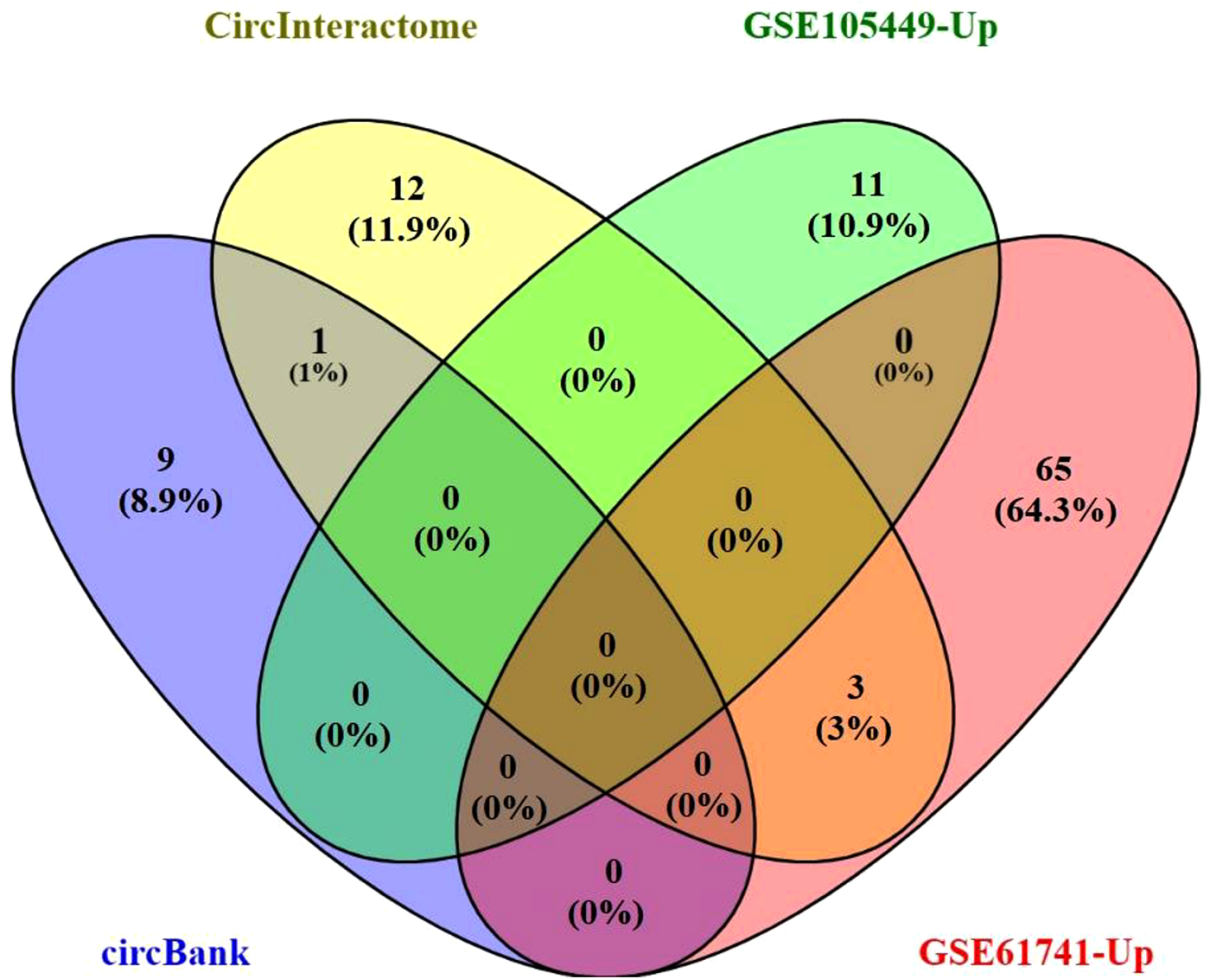
The Venn diagram for the overlap of numbers of the predicted miRNAs.

We used Venny ver. 2.1 to visualize the Circbank and Circinteractome data, the upregulated miRNAs of GSE105449 and GSE61741 dataset; and their intersections (Figure 3). The predicted target miRNAs of hsa_circ_0001445 were hsa-miR-507, hsa-miR-375–3p, hsa-miR-576–5p, and hsa-miR-942–5p. These four miRNAs were entered into Cytoscape ver. 3.6.1. The CyTargetLinker app simply links Cytoscape networks to miRNA-mRNA interactions. A total of 1,175 mRNAs were obtained and the Gene IDs were converted into Entrez IDs for GO and KEGG analyses using R software and the Perl tool.

### GO and KEGG analysis

**Figure 4:**
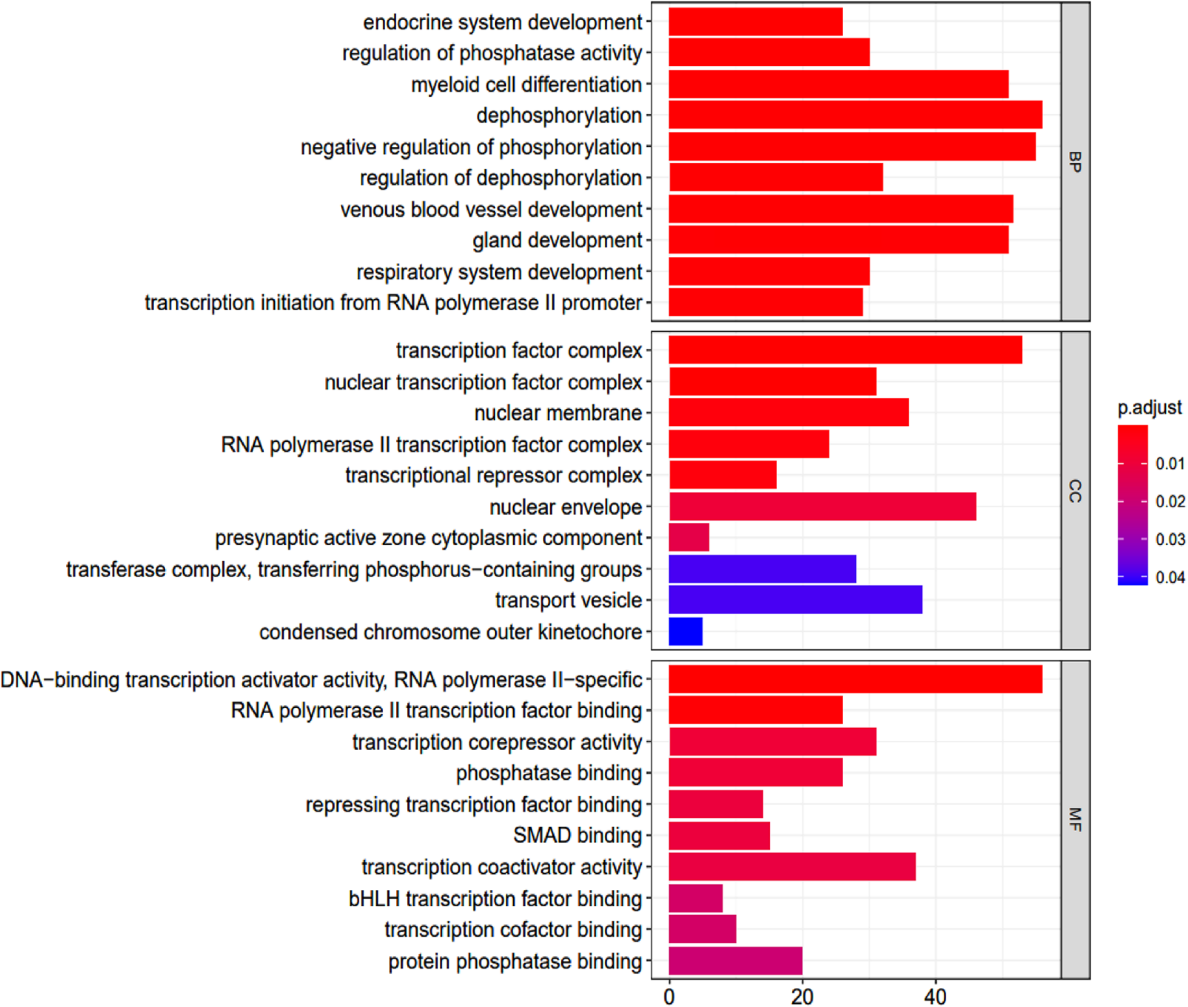
Go enrichment analysis. BP, biological process; CC, cellular component; MF, molecular function.

GO terms were analyzed using R ver. 3.6.2 and clusterProfiler ver. 3.14.3 followed by pathway enrichment. A total of 405 GO terms were found (Supplementary Table S2). These covered molecular function pathways, as shown in the table. The GO terms were ranked by their adjusted *p*-values (*p*-value <0.05; *p*-adjusted <0.05) (Figure 4).

**Figure 5:**
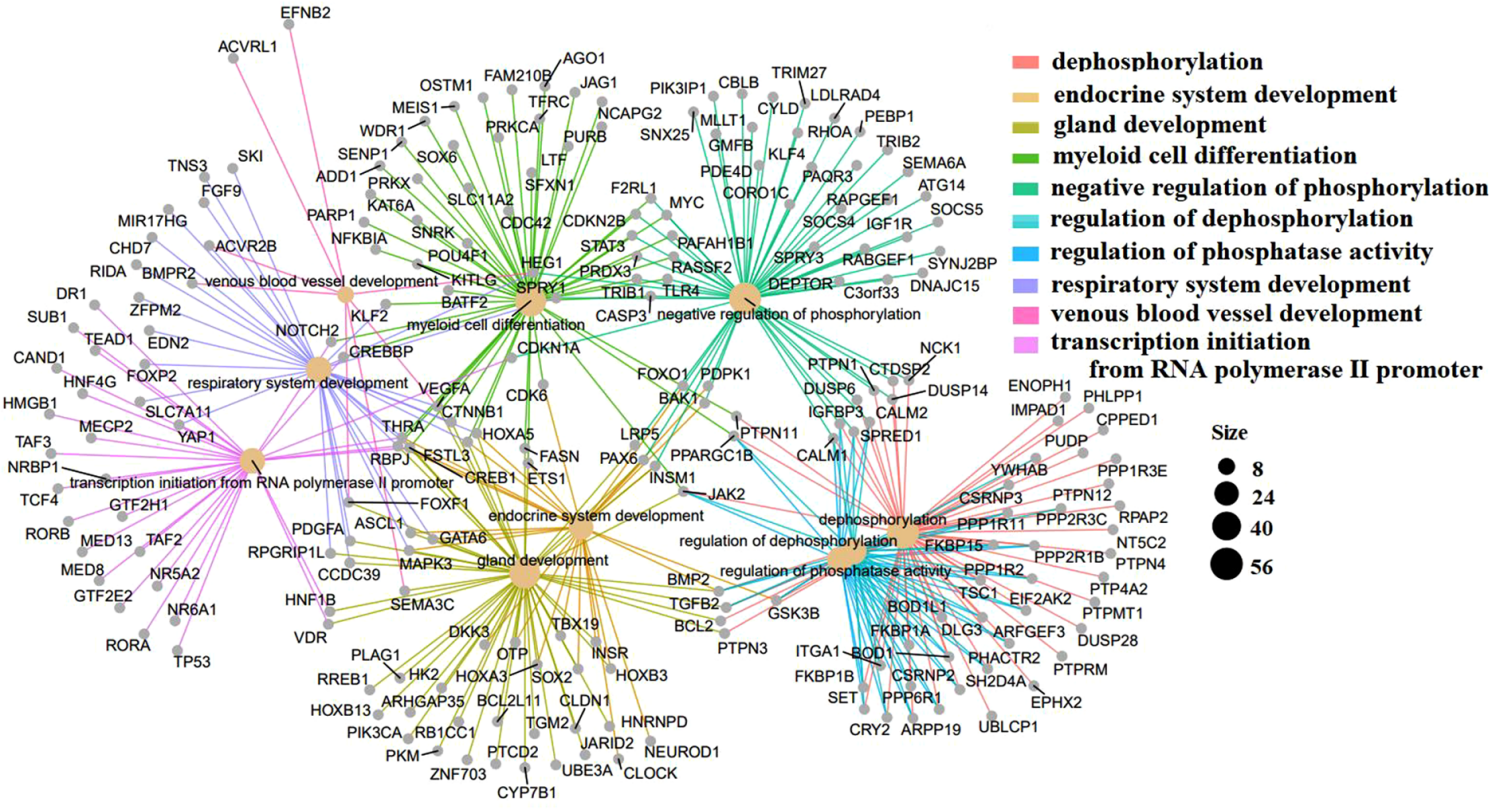
Top 10 GO terms with the lowest *p*-values. Biological pathways and associated genes were identified. Each node indicates a biological process in the path. The lines are the interactions between the genes and the terms.

The GO terms indicated biological process pathways such as dephosphorylation, negative regulation of phosphorylation, and venous blood vessel development (Figure 5).

**Figure 6:**
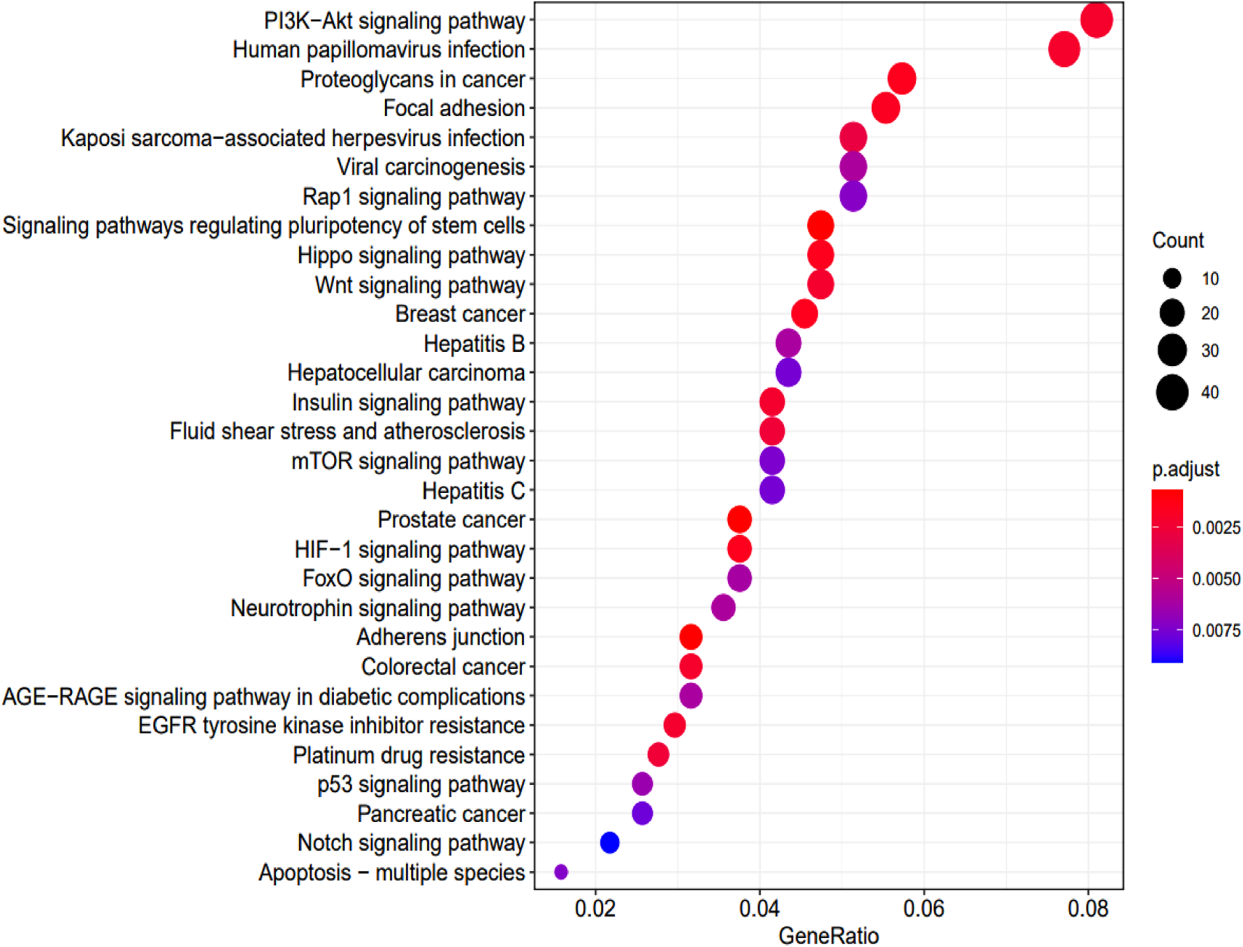
KEGG pathway analysis and the numbers of associated genes. The pathways and the associated genes were ranked based on the *p*-values. The sizes of the dots represent the gene counts of each row (KEGG categories).

KEGG enrichment analysis identified 70 pathways (*p*-values <0.05) (Supplementary Table S3). The top 30 are summarized in Figure 6.

**Figure 7:**
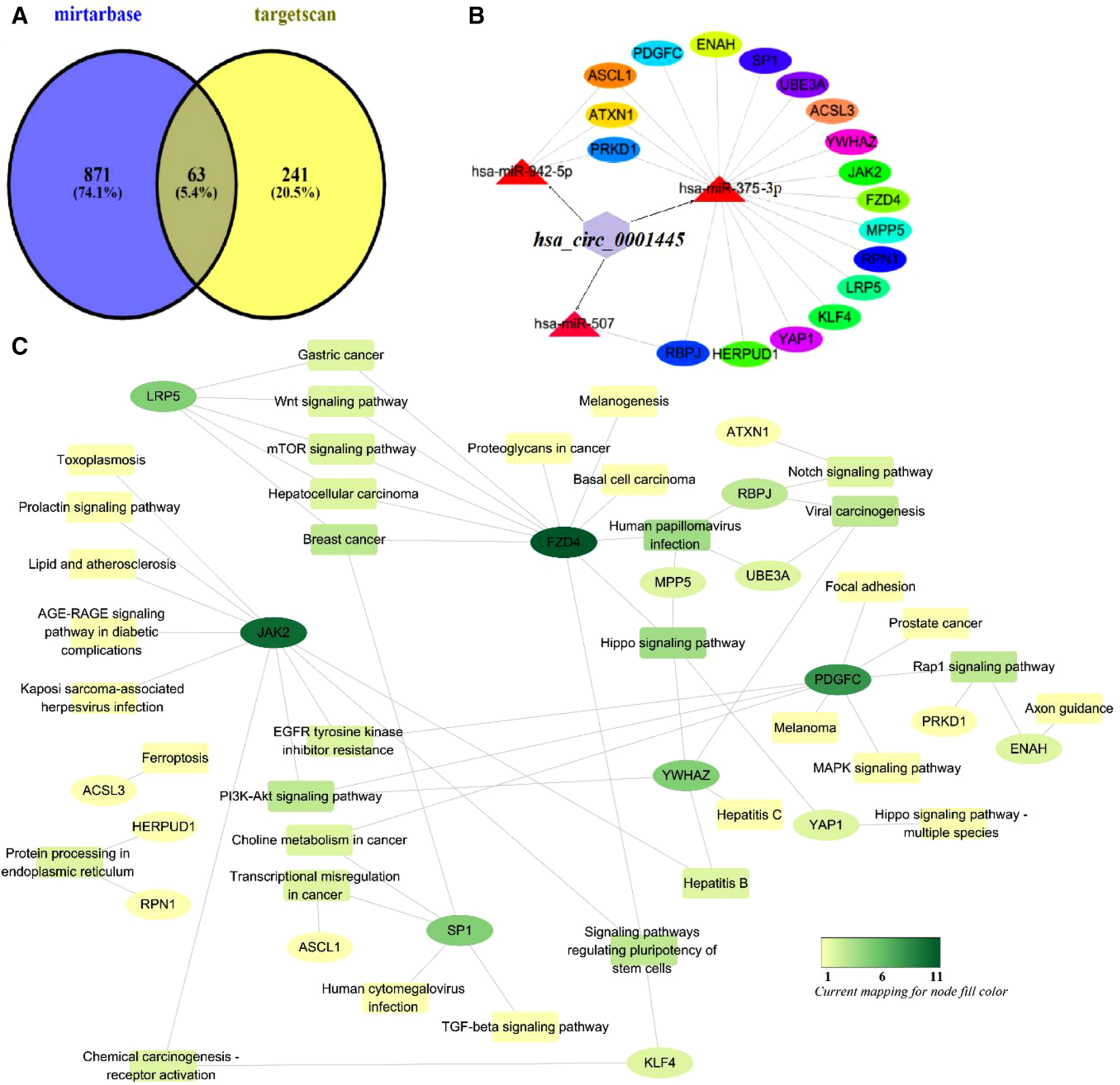
Mechanism of action of hsa_circ_0001445. (**A**) Venn diagram of genes predicted by mirtarbase and targetscan. (**B**) The subnetwork of the interactions between miRNAs and these genes. (**C**) Functional analysis of potential genes of the KEGG pathway using Cytoscape ver. 3.6.1.

To enhance accuracy, the results afforded by the mirtarbase and targetscan databases were intersected with those described above (Figure 7). This yielded 63 genes that were compared to KEGG pathway genes. We found 18 potential KEGG genes. Finally, we built a subnetwork of the interactions between miRNAs and these genes. This included the signaling pathways mentioned.

## Discussion

Identifying factors involved in CHD pathogenesis can not only improves our understanding of its development but also suggests new approaches to the diagnosis, prognosis, and management of CHD. Numerous biomarkers associated with CHD have been applied, however, to identify its primary stage by regular examinations, such as cardiac ultrasound and electrocardiography, remains challenging. CHD diagnosis in its early stage with a sensitive biomarker are crucial for treatment and prognosis. Recent large-scale studies have suggested that circRNAs play an essential role in the pathogenesis and progression of CHD (5, 6, 12, 23). In this study, we identified for the first time the circRNA hsa_circ_0001445 as a potential diagnostic biomarker for CHD. By evaluating the expression level of hsa_circ_0001445 in CHD patients and control group, our results showed that the expression of hsa_circ_0001445 in PBLs of CHD patients was significantly lower than that of healthy controls. ROC curve analysis indicated that its expression level well-distinguished CHD patients from healthy subjects with AUC valued 0.816 ± 0.028 (95% CI 0.761–0.871; *p* < 0.001). In addition, logistic regression revealed that hsa_circ_0001445 was an independent predictor of CHD. Further studies on hsa_circ_0001445 as well as other circRNAs involved in CHD is required to investigate their potential in CHD diagnosis.

The RNA binding protein Quaking (QKI) promotes circRNA formation (24). QKI was proven to be involved in cell differentiation, apoptosis, proliferation, and migration (25). Interestingly, recent works have shown that QKI affects cardiovascular development and function (26–29), suggesting that QKI may affect the expression of hsa_circ_0001445 in CHD patients and consequently act on the disease progression. Several studies have also indicated that hsa_circ_0001445 may be involved in CHD development. Vilades et al*.* (6) found that plasma levels of hsa_circ_0001445 are lower in patients with higher coronary atherosclerotic burdens. Moreover, hsa_circ_0001445 is expressed by human coronary smooth muscle cells *in vitro*; its secretion is reduced under atherosclerotic conditions (6). Cai et al*.* (30) found that hsa_circ_0001445 is downregulated during low lipoprotein-induced oxidation of human umbilical vein endothelial cells. Overexpression of hsa_circ_0001445 promotes cell proliferation and inhibits the inflammatory response and apoptosis. These findings and our work suggest that hsa_circ_0001445 may involve in CHD development. Since the molecular mechanism of CHD is complicated, as is the effect of hsa_circ_0001445 on CHD, further research is required to elucidate its role in CHD pathogenesis.

The circRNA-miRNA-mRNA axis has been recently researched in terms of how circRNAs regulate CHD development. Lin et al*.* (31) constructed a network graph of the correlations between hsa-miR-101–5 and each of hsa_circ_0030769, hsa_circ_15486–161, hsa_circ_0122274, and hsa_circ_0079828. Miao et al*.* (5) hypothesized that the miRNAs regulated by hsa_circ_0016274 are miRNA-361–5p, miR-21–3p, miRNA-296–3p, and miRNA-375. The target genes of the circ-YOD1-miR-21–3p/miR-296–3p axis are *BCL6*, *FBXL18*, *MMP9*, and *FCGR3B*, as confirmed in other studies describing associations of *MMP9*, *BCL6* (32, 33), hsa-miR-21–3p (32), and hsa-miR-296–3p (34) with CHD. In another study, the network of circ_ZNF609-related miRNAs included hsa-miR-615–5p, hsa-miR-145–5p, hsa-miR-138–5p, hsa-miR-150–5p (35), and AKT1 (a downstream target of miR-138–5p) (36). In this study, we predicted that certain genes affect CHD through bioinformatics analysis. This finding is in agreement with previous reports as *JAK2* (37, 38), *FZD4* (39), *PDGFC* (40), *YWHAZ* (41), *SP1* (42), and *LRP5* (43–45) which are all targets of hsa-miR-375–3p (46, 47). These genes participate in many signaling pathways, of which the PI3K-Akt pathway may play a major role in CHD (48–52). Phosphoinositide 3-kinase (PI3K) lies downstream of many receptor tyrosine kinases. PI3Ks play crucial roles in many aspects of biological response, such as membrane trafficking, cytoskeletal organization, cell growth and apoptosis. A serine/threonine kinase Akt, also known as protein kinase B, is the most well characterized target of PI3K. Akt is known to mediate cell survival signal by regulating several effectors, increases the rate of initiation of translation of mRNA by ribosomes such as Bad or procaspase-9, p70S6K. Stimulating and activating the phosphoinositide 3-kinase (PI3K)/protein kinase B (Akt) signaling pathway can regulate the expression of vascular endothelial cytokines, the polarization and survival of macrophages, the expression of inflammatory factors and platelet function, thus affecting the occurrence and development of CHD. This reinforces the authenticity of our interactive network prediction. The integrated biological information predicts the potential molecular mechanism of hsa_circ_0001445.

This study had certain limitations. First, it was a cross-sectional study with a modest sample size. Our findings require confirmation in larger studies to obtain higher reliability. Second, we did not perform luciferase assay, WB assay; but only database-derived links. Those binding assays would reinforce our suggestion that hsa_circ_0001445 is a good candidate biomarker of CHD. The mechanism by which the circRNA-miRNA-mRNA axis regulates CHD pathogenesis requires further *in vivo* and *in vitro* research.

## Conclusion

We identified hsa_circ_0001445 as a potential biomarker for CHD diagnosis. The predicted genes involved in CHD participate in many signaling pathways, of which PI3K-Akt signaling may be particularly relevant to CHD. Our results provide a basis for further research on the molecular mechanism of hsa_circ_0001445 in CHD pathogenesis.

## Data Availability

The original contributions presented in the study are included in the article/**Supplementary Material**, further inquiries can be directed to the corresponding author/s.

## Supplementary material

Supplementary Table 1 The analysis for differentiallyexpressed miRNAs.

Supplementary Table 2 The analysis for Gene Ontology.

Supplementary Table 3 The analysis for KEGG pathway.
