# Supplementary material for: Identification of hsa_circ_0001445 of a novel circRNA-miRNA-mRNA regulatory network as potential biomarker for coronary heart disease
Source: Front Cardiovasc Med. 2023 Mar 14;10:1104223. doi: 10.3389/fcvm.2023.1104223 (PMC10043405; doi:10.3389/fcvm.2023.1104223)
Supplement: Supplementary Table 1 — The analysis for differentiallyexpressed miRNAs. [file Table1.docx]

The differentially expressed miRNAs analyses revealed 11 up-regulated and 21 down-regulated miRNAs from GSE105449

| **Up-regulated miRNA** | **log2FC** | **P value** | **Down-regulated miRNA** | **log2FC** | **P value** |
| --- | --- | --- | --- | --- | --- |
| hsa-miR-124-3p | 1.344234242 | 0.008875452 | hsa-miR-186-5p | -1.197982753 | 0.007514646 |
| hsa-miR-1288-3p | 1.175015237 | 0.016246456 | hsa-miR-30b-5p | -1.178711234 | 0.004173543 |
| hsa-miR-139-3p | 1.104053912 | 0.002214564 | hsa-miR-376a-3p | -1.251881245 | 0.040091924 |
| hsa-miR-520e | 1.055113965 | 0.021147573 | hsa-miR-338-3p | -1.156623434 | 0.029673451 |
| hsa-miR-644a | 1.034448666 | 0.005345673 | hsa-miR-326 | -1.146023456 | 0.020356346 |
| hsa-miR-378a-3p | 1.128869631 | 0.031397457 | hsa-miR-29c-5p | -1.118398224 | 0.022234453 |
| hsa-miR-1305 | 1.240584333 | 0.001566456 | hsa-miR-548am-5p | -1.108357546 | 0.010322435 |
| hsa-miR-542-3p | 1.174828635 | 0.008972344 | hsa-miR-29c-3p | -1.104433632 | 0.005536363 |
| hsa-miR-1202 | 1.162524856 | 0.016565743 | hsa-miR-17-5p | -1.094272355 | 0.007023452 |
| hsa-miR-27b-3p | 1.116581263 | 0.021416462 | hsa-miR-335-3p | -1.088737452 | 0.006563467 |
| hsa-miR-204-5p | 1.053371845 | 0.039343546 | hsa-miR-1 | -1.087919764 | 0.027412352 |
|  |  |  | hsa-miR-664-5p | -1.086066784 | 0.029941134 |
|  |  |  | hsa-miR-493-5p | -1.079992345 | 0.003943463 |
|  |  |  | hsa-miR-543 | -1.076457456 | 0.001012523 |
|  |  |  | hsa-miR-30c-5p | -1.071472523 | 0.003167455 |
|  |  |  | hsa-miR-92a-1-5p | -1.052894564 | 0.030275523 |
|  |  |  | hsa-miR-548g-3p | -1.051862342 | 0.045585342 |
|  |  |  | hsa-miR-106b-3p | -1.040316367 | 0.005695345 |
|  |  |  | hsa-miR-624-3p | -1.037265552 | 0.021139553 |
|  |  |  | hsa-miR-28-3p | -1.027932342 | 0.046199513 |
|  |  |  | hsa-miR-548c-3p | -1.003078345 | 0.037346394 |

The differentially expressed miRNAs analyses revealed 68 up-regulated and 32 down-regulated miRNAs from GSE61741

| **Up-regulated miRNA** | **log2FC** | **P value** | **Down-regulated miRNA** | **log2FC** | **P value** |
| --- | --- | --- | --- | --- | --- |
| hsa-miR-375-3p | 2.055179668 | 3.41E-08 | hsa-miR-31-3p | -1.67133085 | 9.10E-08 |
| hsa-miR-142-3p | 1.911637447 | 5.52E-07 | hsa-miR-1283 | -1.632062369 | 8.10E-07 |
| hsa-miR-29c-3p | 1.850096182 | 4.86E-08 | hsa-miR-200a | -1.60499372 | 3.77E-07 |
| hsa-miR-1258 | 1.846107258 | 1.34E-07 | hsa-miR-515-5p | -1.467324166 | 4.04E-08 |
| hsa-miR-302b-3p | 1.789940606 | 2.09E-06 | hsa-miR-1245a | -1.409488453 | 3.83E-06 |
| hsa-miR-1468-5p | 1.711611027 | 1.76E-06 | hsa-miR-155-3p | -1.376257877 | 1.03E-05 |
| hsa-miR-520c-3p | 1.647465652 | 7.74E-07 | hsa-miR-488-5p | -1.368851066 | 3.19E-07 |
| hsa-miR-204-5p | 1.643870075 | 7.73E-07 | hsa-miR-21-3p | -1.324586005 | 0.000113238 |
| hsa-miR-609 | 1.621093043 | 2.97E-05 | hsa-miR-519b-5p | -1.275137605 | 9.00E-06 |
| hsa-miR-132-5p | 1.603354363 | 1.14E-07 | hsa-miR-545-3p | -1.217793064 | 8.00E-05 |
| hsa-miR-601 | 1.56530189 | 8.80E-06 | hsa-miR-518d-5p | -1.208109894 | 0.000309135 |
| hsa-miR-1282 | 1.514761201 | 0.000481865 | hsa-miR-520d-5p | -1.141628173 | 0.000143336 |
| hsa-miR-942-5p | 1.511218368 | 4.60E-05 | hsa-miR-491-3p | -1.12805534 | 0.000296522 |
| hsa-miR-508-3p | 1.490992456 | 3.70E-05 | hsa-miR-136 | -1.124245173 | 0.000120221 |
| hsa-miR-190b | 1.484957486 | 0.00015187 | hsa-miR-564 | -1.124118948 | 0.000172726 |
| hsa-miR-488 | 1.457383303 | 7.14E-05 | hsa-miR-450b-5p | -1.11582486 | 0.000412086 |
| hsa-miR-522 | 1.417918024 | 5.99E-05 | hsa-miR-589 | -1.113168964 | 9.50E-05 |
| hsa-miR-483-3p | 1.406653152 | 0.000108236 | hsa-miR-568 | -1.092523865 | 0.0003474 |
| hsa-miR-135b | 1.39903074 | 4.57E-05 | hsa-miR-566 | -1.091148269 | 2.44E-06 |
| hsa-miR-154 | 1.39552332 | 2.82E-05 | hsa-miR-489 | -1.080053973 | 1.62E-06 |
| hsa-miR-578 | 1.341549259 | 0.000398976 | hsa-miR-646 | -1.075597728 | 4.17E-06 |
| hsa-miR-98 | 1.339550111 | 0.000705281 | hsa-miR-96 | -1.062896733 | 0.00029237 |
| hsa-miR-302d | 1.3359602 | 3.28E-05 | hsa-miR-889 | -1.061404435 | 0.001308786 |
| hsa-miR-373 | 1.317050802 | 6.99E-05 | hsa-miR-518d-3p | -1.058502452 | 0.00117974 |
| hsa-miR-520d-3p | 1.283811893 | 0.00706914 | hsa-miR-518a-5p | -1.039219331 | 5.40E-05 |
| hsa-miR-199b-5p | 1.276995292 | 0.000134431 | hsa-miR-10b | -1.038068175 | 0.000632201 |
| hsa-miR-576-5p | 1.275121454 | 1.22E-05 | hsa-miR-933 | -1.036233491 | 0.000205256 |
| hsa-miR-126 | 1.274789058 | 9.37E-05 | hsa-miR-31 | -1.025541714 | 3.18E-05 |
| hsa-miR-450a | 1.272068258 | 0.000490697 | hsa-miR-556-5p | -1.019093938 | 0.001029336 |
| hsa-miR-382 | 1.271701427 | 0.000873747 | hsa-miR-1291 | -1.007853696 | 1.53E-05 |
| hsa-miR-520f | 1.236694357 | 0.000275999 | hsa-miR-298 | -1.000913151 | 0.001879195 |
| hsa-miR-556-3p | 1.235964409 | 0.000729576 | hsa-miR-455-3p | -1.000870625 | 2.97E-06 |
| hsa-miR-196a | 1.213652697 | 0.001077973 |  |  |  |
| hsa-miR-1238 | 1.210567371 | 0.000888843 |  |  |  |
| hsa-miR-1299 | 1.208585133 | 0.000638899 |  |  |  |
| hsa-miR-581 | 1.206230568 | 0.002589965 |  |  |  |
| hsa-let-7b | 1.197792319 | 0.000592376 |  |  |  |
| hsa-miR-133a | 1.196438055 | 7.17E-05 |  |  |  |
| hsa-miR-26b | 1.192595412 | 0.000812463 |  |  |  |
| hsa-miR-26a-2 | 1.165144202 | 0.000394472 |  |  |  |
| hsa-miR-644 | 1.164675382 | 0.001061778 |  |  |  |
| hsa-miR-876-3p | 1.1633107 | 0.000842051 |  |  |  |
| hsa-miR-1262 | 1.146834711 | 0.000111841 |  |  |  |
| hsa-miR-1290 | 1.142965882 | 0.000285906 |  |  |  |
| hsa-miR-629 | 1.131519344 | 0.001971544 |  |  |  |
| hsa-miR-892b | 1.122018517 | 0.000413561 |  |  |  |
| hsa-miR-29b-1 | 1.121534449 | 0.000395941 |  |  |  |
| hsa-miR-613 | 1.120859144 | 0.002689712 |  |  |  |
| hsa-miR-23a | 1.118564526 | 0.001649144 |  |  |  |
| hsa-miR-1246 | 1.106920355 | 0.001925949 |  |  |  |
| hsa-miR-330-5p | 1.100359291 | 0.001162245 |  |  |  |
| hsa-miR-335 | 1.0982608 | 0.002582637 |  |  |  |
| hsa-miR-509-3p | 1.096876194 | 0.001752571 |  |  |  |
| hsa-miR-34a | 1.065632823 | 0.001566126 |  |  |  |
| hsa-let-7f-2 | 1.06162993 | 0.002111737 |  |  |  |
| hsa-miR-302a | 1.05910759 | 0.002523253 |  |  |  |
| hsa-miR-1825 | 1.044613349 | 0.000424001 |  |  |  |
| hsa-miR-615-3p | 1.04148496 | 0.00045637 |  |  |  |
| hsa-miR-455-5p | 1.041114067 | 0.004762951 |  |  |  |
| hsa-miR-454 | 1.040488361 | 0.002137958 |  |  |  |
| hsa-miR-1279 | 1.037165803 | 0.000687152 |  |  |  |
| hsa-miR-9 | 1.036982227 | 0.00486468 |  |  |  |
| hsa-miR-130b | 1.024452525 | 0.000302821 |  |  |  |
| hsa-miR-515-3p | 1.023071493 | 0.002013029 |  |  |  |
| hsa-miR-133b | 1.021365316 | 0.002489713 |  |  |  |
| hsa-miR-376a | 1.005936915 | 0.003021089 |  |  |  |
| hsa-miR-340 | 1.004367358 | 0.002193518 |  |  |  |
| hsa-miR-598 | 1.000709176 | 2.42E-05 |  |  |  |
